# Benzyl *N*′-(1*H*-indol-2-ylmethyl­ene)hydrazinecarbodithio­ate ethanol hemisolvate

**DOI:** 10.1107/S1600536808038609

**Published:** 2008-11-26

**Authors:** Hamid Khaledi, Hapipah Mohd Ali, Seik Weng Ng

**Affiliations:** a Department of Chemistry, University of Malaya, 50603 Kuala Lumpur, Malaysia

## Abstract

In the crystal of the title compound, C_17_H_15_N_3_S_2_·0.5C_2_H_6_O, the mol­ecules are linked by a pair of N—H_aliphatic_⋯S hydrogen bonds across a center of inversion, forming a dimer. The ethanol solvent mol­ecule, which is statistically disordered about a crystallographic twofold rotation axis, accepts an N—H_aromatic_⋯O hydrogen bond; the hydr­oxy group of the solvent mol­ecule is not engaged in hydrogen bonding.

## Related literature

For references to benzyl esters of hydrazinecarbodithioic acids, see: Khaledi *et al.* (2008 ▶).
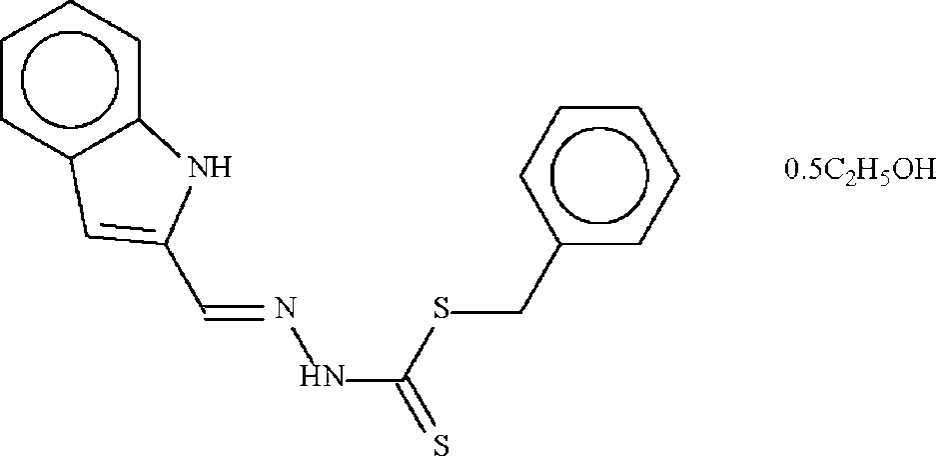


## Experimental

### 

#### Crystal data

C_17_H_15_N_3_S_2_·0.5C_2_H_6_O
*M* *_r_* = 348.47
Monoclinic, 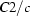

*a* = 13.4225 (2) Å
*b* = 15.4088 (2) Å
*c* = 16.8120 (3) Å
β = 102.637 (1)°
*V* = 3392.90 (9) Å^3^
*Z* = 8
Mo *K*α radiation
μ = 0.32 mm^−1^
*T* = 100 (2) K
0.25 × 0.20 × 0.15 mm

#### Data collection

Bruker SMART APEX CCD diffractometer
Absorption correction: multi-scan (*SADABS*; Sheldrick, 1996 ▶) *T*_min_ = 0.924, *T*_max_ = 0.954
14302 measured reflections
3897 independent reflections
3332 reflections with *I* > 2σ(*I*)
*R*_int_ = 0.028

#### Refinement

*R*[*F* ^2^ > 2σ(*F* ^2^)] = 0.033
*wR*(*F* ^2^) = 0.088
*S* = 1.03
3897 reflections
232 parameters
5 restraints
H atoms treated by a mixture of independent and constrained refinement
Δρ_max_ = 0.35 e Å^−3^
Δρ_min_ = −0.40 e Å^−3^

### 

Data collection: *APEX2* (Bruker, 2007 ▶); cell refinement: *SAINT* (Bruker, 2007 ▶); data reduction: *SAINT*; program(s) used to solve structure: *SHELXS97* (Sheldrick, 2008 ▶); program(s) used to refine structure: *SHELXL97* (Sheldrick, 2008 ▶); molecular graphics: *X-SEED* (Barbour, 2001 ▶); software used to prepare material for publication: *pubCIF* (Westrip, 2008 ▶).

## Supplementary Material

Crystal structure: contains datablocks global, I. DOI: 10.1107/S1600536808038609/hb2851sup1.cif

Structure factors: contains datablocks I. DOI: 10.1107/S1600536808038609/hb2851Isup2.hkl

Additional supplementary materials:  crystallographic information; 3D view; checkCIF report

## Figures and Tables

**Table 1:** Hydrogen-bond geometry (Å, °)

*D*—H⋯*A*	*D*—H	H⋯*A*	*D*⋯*A*	*D*—H⋯*A*
N1—H1*n*⋯S2^i^	0.88 (1)	2.52 (1)	3.350 (1)	159 (2)
N3—H3*n*⋯O1	0.87 (1)	2.25 (1)	3.047 (3)	153 (2)

Symmetry code: (i) 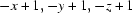.

**Fig. 1.**
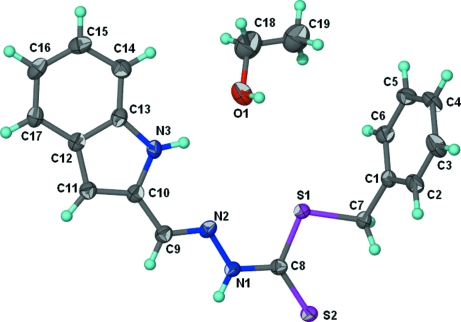
View of (I) at the 70% probability level. Hydrogen atoms are drawn as spheres of arbitrary radius. Only one orientation of the ethanol molecule is shown.

## supplementary crystallographic information

### Comment

For background references, see: Khaledi *et al.* (2008).

### Experimental

Indole-2-carbaldehyde (0.36 g, 2.5 mmol) and *S*-benzyldithiocarbazate (0.495 g, 2.5 mmol) were heated in ethanol (40 ml) for 3 h. The solution was set aside for the formation of yellow blocks of (I).

### Refinement

C-bound hydrogen atoms were placed at calculated positions (C–H = 0.95–0.99 Å) and refined as riding with *U*(H) = 1.2–1.5 times *U*_eq_(C). The amino and hydroxy H atoms were located in a difference map, and were refined with distance restraints of N–H = 0.88±0.01 and O–H = 0.84+_0.01 Å; their U_iso_ values were freely refined.

The ethanol molecule is statistically disordered about a two-fold axis: the O–C distance was restrained to 1.45±0.01 Å and the C–C distance to 1.50+_0.01 Å; the displacement factors of the two C atoms were restrained to be equal.

### Crystal data

**Table:**

C_17_H_15_N_3_S_2_·0.5C_2_H_6_O	*F*_000_ = 1464
*M**_r_* = 348.47	*D*_x_ = 1.364 Mg m^−^^3^
Monoclinic, *C*2/*c*	Mo *K*α radiation λ = 0.71073 Å
Hall symbol: -C 2yc	Cell parameters from 5259 reflections
*a* = 13.4225 (2) Å	θ = 2.5–28.3º
*b* = 15.4088 (2) Å	µ = 0.32 mm^−^^1^
*c* = 16.8120 (3) Å	*T* = 100 (2) K
β = 102.637 (1)º	Block, yellow
*V* = 3392.90 (9) Å^3^	0.25 × 0.20 × 0.15 mm
*Z* = 8	

### Data collection

**Table:**

Bruker SMART APEX CCD diffractometer	3897 independent reflections
Radiation source: fine-focus sealed tube	3332 reflections with *I* > 2σ(*I*)
Monochromator: graphite	*R*_int_ = 0.028
*T* = 100(2) K	θ_max_ = 27.5º
ω scans	θ_min_ = 2.0º
Absorption correction: multi-scan(SADABS; Sheldrick, 1996)	*h* = −16→17
*T*_min_ = 0.924, *T*_max_ = 0.954	*k* = −19→20
14302 measured reflections	*l* = −21→17

### Refinement

**Table:**

Refinement on *F*^2^	Secondary atom site location: difference Fourier map
Least-squares matrix: full	Hydrogen site location: inferred from neighbouring sites
*R*[*F*^2^ > 2σ(*F*^2^)] = 0.033	H atoms treated by a mixture of independent and constrained refinement
*wR*(*F*^2^) = 0.088	*w* = 1/[σ^2^(*F*_o_^2^) + (0.0411*P*)^2^ + 3.3308*P*] where *P* = (*F*_o_^2^ + 2*F*_c_^2^)/3
*S* = 1.03	(Δ/σ)_max_ = 0.001
3897 reflections	Δρ_max_ = 0.35 e Å^−^^3^
232 parameters	Δρ_min_ = −0.40 e Å^−^^3^
5 restraints	Extinction correction: none
Primary atom site location: structure-invariant direct methods	

### Fractional atomic coordinates and isotropic or equivalent isotropic displacement parameters (Å^2^)

**Table:**

	*x*	*y*	*z*	*U*_iso_*/*U*_eq_	Occ. (<1)
S1	0.58648 (3)	0.69507 (2)	0.36508 (2)	0.01972 (10)	
S2	0.56141 (3)	0.50160 (2)	0.38844 (2)	0.02263 (11)	
N1	0.48149 (10)	0.62574 (8)	0.46106 (8)	0.0201 (3)	
H1N	0.4612 (15)	0.5843 (10)	0.4893 (11)	0.036 (5)*	
N2	0.45977 (10)	0.71147 (8)	0.47237 (8)	0.0200 (3)	
N3	0.39716 (10)	0.88130 (8)	0.48940 (8)	0.0205 (3)	
H3N	0.4341 (14)	0.8771 (13)	0.4528 (10)	0.039 (6)*	
C1	0.72885 (12)	0.71544 (9)	0.27832 (9)	0.0199 (3)	
C2	0.83286 (12)	0.71386 (11)	0.31290 (10)	0.0254 (3)	
H2	0.8608	0.6671	0.3476	0.031*	
C3	0.89612 (13)	0.77961 (12)	0.29733 (10)	0.0297 (4)	
H3	0.9671	0.7776	0.3212	0.036*	
C4	0.85687 (13)	0.84829 (11)	0.24721 (10)	0.0260 (3)	
H4	0.9004	0.8937	0.2372	0.031*	
C5	0.75366 (13)	0.85010 (10)	0.21189 (10)	0.0242 (3)	
H5	0.7263	0.8968	0.1770	0.029*	
C6	0.68972 (12)	0.78405 (9)	0.22712 (10)	0.0222 (3)	
H6	0.6190	0.7858	0.2024	0.027*	
C7	0.66018 (12)	0.64525 (9)	0.29845 (10)	0.0211 (3)	
H7A	0.7011	0.5962	0.3264	0.025*	
H7B	0.6144	0.6232	0.2482	0.025*	
C8	0.53949 (11)	0.60480 (9)	0.40842 (9)	0.0180 (3)	
C9	0.40256 (11)	0.72590 (9)	0.52304 (9)	0.0203 (3)	
H9	0.3811	0.6794	0.5524	0.024*	
C10	0.37149 (11)	0.81355 (9)	0.53466 (9)	0.0197 (3)	
C11	0.31225 (12)	0.84426 (9)	0.58549 (9)	0.0208 (3)	
H11	0.2848	0.8111	0.6233	0.025*	
C12	0.29954 (11)	0.93534 (9)	0.57094 (9)	0.0192 (3)	
C13	0.35401 (11)	0.95615 (9)	0.51045 (9)	0.0197 (3)	
C14	0.35929 (12)	1.04048 (10)	0.48156 (10)	0.0246 (3)	
H14	0.3960	1.0533	0.4407	0.030*	
C15	0.30886 (13)	1.10461 (10)	0.51477 (10)	0.0269 (4)	
H15	0.3116	1.1628	0.4967	0.032*	
C16	0.25378 (13)	1.08549 (10)	0.57456 (10)	0.0259 (3)	
H16	0.2196	1.1309	0.5960	0.031*	
C17	0.24827 (12)	1.00197 (10)	0.60284 (10)	0.0229 (3)	
H17	0.2105	0.9897	0.6431	0.027*	
O1	0.48913 (18)	0.92618 (16)	0.34417 (14)	0.0289 (5)	0.50
H1O	0.5523 (10)	0.922 (3)	0.363 (3)	0.045 (13)*	0.50
C18	0.4709 (7)	1.0002 (6)	0.2875 (4)	0.0472 (9)	0.50
H18A	0.3966	1.0099	0.2687	0.057*	0.50
H18B	0.5016	1.0533	0.3157	0.057*	0.50
C19	0.5162 (7)	0.9824 (6)	0.2172 (4)	0.0472 (9)	0.50
H19A	0.5044	1.0320	0.1798	0.071*	0.50
H19B	0.4847	0.9304	0.1888	0.071*	0.50
H19C	0.5898	0.9729	0.2360	0.071*	0.50

### Atomic displacement parameters (Å^2^)

**Table:**

	*U*^11^	*U*^22^	*U*^33^	*U*^12^	*U*^13^	*U*^23^
S1	0.0245 (2)	0.01412 (17)	0.0230 (2)	0.00081 (13)	0.01047 (15)	0.00013 (13)
S2	0.0342 (2)	0.01388 (17)	0.0233 (2)	−0.00028 (14)	0.01385 (17)	−0.00209 (14)
N1	0.0271 (7)	0.0148 (6)	0.0210 (7)	0.0021 (5)	0.0108 (5)	0.0004 (5)
N2	0.0225 (6)	0.0171 (6)	0.0203 (7)	0.0026 (5)	0.0042 (5)	−0.0024 (5)
N3	0.0223 (6)	0.0210 (6)	0.0204 (7)	0.0009 (5)	0.0092 (5)	−0.0024 (5)
C1	0.0255 (8)	0.0185 (7)	0.0183 (7)	−0.0008 (6)	0.0104 (6)	−0.0024 (5)
C2	0.0261 (8)	0.0299 (8)	0.0217 (8)	0.0038 (6)	0.0082 (7)	0.0082 (6)
C3	0.0207 (8)	0.0439 (10)	0.0241 (9)	−0.0035 (7)	0.0045 (7)	0.0074 (7)
C4	0.0293 (9)	0.0299 (8)	0.0202 (8)	−0.0086 (7)	0.0087 (7)	0.0026 (6)
C5	0.0316 (9)	0.0207 (7)	0.0204 (8)	−0.0001 (6)	0.0062 (7)	0.0028 (6)
C6	0.0229 (8)	0.0213 (7)	0.0222 (8)	−0.0002 (6)	0.0044 (6)	−0.0009 (6)
C7	0.0258 (8)	0.0179 (7)	0.0221 (8)	0.0001 (6)	0.0110 (6)	−0.0025 (6)
C8	0.0199 (7)	0.0170 (6)	0.0169 (7)	−0.0003 (5)	0.0031 (6)	0.0001 (5)
C9	0.0224 (7)	0.0199 (7)	0.0182 (7)	0.0022 (6)	0.0038 (6)	−0.0003 (6)
C10	0.0199 (7)	0.0209 (7)	0.0178 (7)	0.0013 (6)	0.0028 (6)	−0.0012 (6)
C11	0.0242 (8)	0.0206 (7)	0.0181 (8)	0.0021 (6)	0.0057 (6)	−0.0003 (6)
C12	0.0184 (7)	0.0217 (7)	0.0164 (7)	0.0012 (6)	0.0014 (6)	−0.0025 (6)
C13	0.0187 (7)	0.0209 (7)	0.0190 (8)	0.0007 (6)	0.0032 (6)	−0.0043 (6)
C14	0.0281 (8)	0.0227 (7)	0.0235 (8)	−0.0033 (6)	0.0066 (7)	−0.0018 (6)
C15	0.0333 (9)	0.0183 (7)	0.0270 (9)	0.0001 (6)	0.0017 (7)	−0.0020 (6)
C16	0.0265 (8)	0.0234 (7)	0.0262 (9)	0.0053 (6)	0.0020 (7)	−0.0082 (6)
C17	0.0224 (8)	0.0263 (8)	0.0203 (8)	0.0030 (6)	0.0057 (6)	−0.0046 (6)
O1	0.0238 (12)	0.0401 (13)	0.0217 (12)	−0.0033 (10)	0.0026 (10)	0.0035 (10)
C18	0.041 (2)	0.050 (3)	0.0478 (15)	0.0096 (19)	0.0033 (12)	−0.001 (2)
C19	0.041 (2)	0.050 (3)	0.0478 (15)	0.0096 (19)	0.0033 (12)	−0.001 (2)

### Geometric parameters (Å, °)

**Table:**

S1—C8	1.7506 (15)	C9—C10	1.439 (2)
S1—C7	1.8187 (15)	C9—H9	0.9500
S2—C8	1.6647 (14)	C10—C11	1.372 (2)
N1—C8	1.3397 (19)	C11—C12	1.428 (2)
N1—N2	1.3748 (16)	C11—H11	0.9500
N1—H1N	0.875 (9)	C12—C17	1.406 (2)
N2—C9	1.285 (2)	C12—C13	1.413 (2)
N3—C13	1.3713 (18)	C13—C14	1.394 (2)
N3—C10	1.3792 (19)	C14—C15	1.383 (2)
N3—H3N	0.873 (9)	C14—H14	0.9500
C1—C2	1.390 (2)	C15—C16	1.403 (2)
C1—C6	1.392 (2)	C15—H15	0.9500
C1—C7	1.507 (2)	C16—C17	1.379 (2)
C2—C3	1.383 (2)	C16—H16	0.9500
C2—H2	0.9500	C17—H17	0.9500
C3—C4	1.383 (2)	O1—C18	1.472 (8)
C3—H3	0.9500	O1—H1O	0.839 (10)
C4—C5	1.383 (2)	C18—C19	1.469 (5)
C4—H4	0.9500	C18—H18A	0.9900
C5—C6	1.391 (2)	C18—H18B	0.9900
C5—H5	0.9500	C19—H19A	0.9800
C6—H6	0.9500	C19—H19B	0.9800
C7—H7A	0.9900	C19—H19C	0.9800
C7—H7B	0.9900		
			
C8—S1—C7	102.42 (7)	C11—C10—N3	109.34 (13)
C8—N1—N2	119.62 (12)	C11—C10—C9	129.24 (14)
C8—N1—H1N	118.6 (13)	N3—C10—C9	121.37 (14)
N2—N1—H1N	121.7 (13)	C10—C11—C12	107.22 (13)
C9—N2—N1	115.70 (13)	C10—C11—H11	126.4
C13—N3—C10	108.88 (12)	C12—C11—H11	126.4
C13—N3—H3N	125.6 (14)	C17—C12—C13	118.83 (14)
C10—N3—H3N	125.5 (14)	C17—C12—C11	134.62 (15)
C2—C1—C6	118.72 (14)	C13—C12—C11	106.55 (13)
C2—C1—C7	120.08 (14)	N3—C13—C14	129.59 (14)
C6—C1—C7	121.17 (14)	N3—C13—C12	108.01 (13)
C3—C2—C1	120.66 (15)	C14—C13—C12	122.40 (14)
C3—C2—H2	119.7	C15—C14—C13	117.29 (15)
C1—C2—H2	119.7	C15—C14—H14	121.4
C4—C3—C2	120.53 (15)	C13—C14—H14	121.4
C4—C3—H3	119.7	C14—C15—C16	121.36 (15)
C2—C3—H3	119.7	C14—C15—H15	119.3
C3—C4—C5	119.29 (15)	C16—C15—H15	119.3
C3—C4—H4	120.4	C17—C16—C15	121.27 (14)
C5—C4—H4	120.4	C17—C16—H16	119.4
C4—C5—C6	120.45 (15)	C15—C16—H16	119.4
C4—C5—H5	119.8	C16—C17—C12	118.83 (15)
C6—C5—H5	119.8	C16—C17—H17	120.6
C5—C6—C1	120.35 (15)	C12—C17—H17	120.6
C5—C6—H6	119.8	C18—O1—H1O	108 (3)
C1—C6—H6	119.8	C19—C18—O1	109.7 (7)
C1—C7—S1	106.10 (10)	C19—C18—H18A	109.7
C1—C7—H7A	110.5	O1—C18—H18A	109.7
S1—C7—H7A	110.5	C19—C18—H18B	109.7
C1—C7—H7B	110.5	O1—C18—H18B	109.7
S1—C7—H7B	110.5	H18A—C18—H18B	108.2
H7A—C7—H7B	108.7	C18—C19—H19A	109.5
N1—C8—S2	121.15 (11)	C18—C19—H19B	109.5
N1—C8—S1	113.45 (10)	H19A—C19—H19B	109.5
S2—C8—S1	125.40 (9)	C18—C19—H19C	109.5
N2—C9—C10	119.03 (14)	H19A—C19—H19C	109.5
N2—C9—H9	120.5	H19B—C19—H19C	109.5
C10—C9—H9	120.5		
			
C8—N1—N2—C9	179.09 (14)	N2—C9—C10—C11	−179.23 (16)
C6—C1—C2—C3	0.6 (2)	N2—C9—C10—N3	3.6 (2)
C7—C1—C2—C3	−177.57 (15)	N3—C10—C11—C12	0.47 (17)
C1—C2—C3—C4	0.2 (3)	C9—C10—C11—C12	−176.97 (15)
C2—C3—C4—C5	−0.8 (3)	C10—C11—C12—C17	179.98 (17)
C3—C4—C5—C6	0.6 (2)	C10—C11—C12—C13	−0.39 (17)
C4—C5—C6—C1	0.2 (2)	C10—N3—C13—C14	179.53 (16)
C2—C1—C6—C5	−0.8 (2)	C10—N3—C13—C12	0.12 (17)
C7—C1—C6—C5	177.36 (14)	C17—C12—C13—N3	179.86 (13)
C2—C1—C7—S1	106.98 (14)	C11—C12—C13—N3	0.16 (16)
C6—C1—C7—S1	−71.14 (16)	C17—C12—C13—C14	0.4 (2)
C8—S1—C7—C1	−163.14 (11)	C11—C12—C13—C14	−179.30 (14)
N2—N1—C8—S2	−176.00 (11)	N3—C13—C14—C15	−179.11 (15)
N2—N1—C8—S1	3.72 (18)	C12—C13—C14—C15	0.2 (2)
C7—S1—C8—N1	179.96 (11)	C13—C14—C15—C16	−0.6 (2)
C7—S1—C8—S2	−0.33 (12)	C14—C15—C16—C17	0.3 (3)
N1—N2—C9—C10	−176.43 (13)	C15—C16—C17—C12	0.3 (2)
C13—N3—C10—C11	−0.38 (17)	C13—C12—C17—C16	−0.7 (2)
C13—N3—C10—C9	177.30 (13)	C11—C12—C17—C16	178.94 (16)

### Hydrogen-bond geometry (Å, °)

**Table:**

*D*—H···*A*	*D*—H	H···*A*	*D*···*A*	*D*—H···*A*
N1—H1n···S2^i^	0.88 (1)	2.52 (1)	3.350 (1)	159 (2)
N3—H3n···O1	0.87 (1)	2.25 (1)	3.047 (3)	153 (2)

Symmetry codes: (i) −*x*+1, −*y*+1, −*z*+1.
